# Effect of Stearyl Methacrylate Comonomer on the Mechanical and Physical Properties of Dimethacrylate-Based Dental Resins

**DOI:** 10.3390/ma17164136

**Published:** 2024-08-21

**Authors:** Mecit Karadag, Emrah Dolekcekic, Murat Erdem, Mutlu Özcan

**Affiliations:** 1 Department of Materials Science and Engineering, Institution of Graduate Schools, Eskisehir Technical University, 26555 Eskisehir, Turkey; 2 Department of Metallurgy, Vocational High School, Bilecik Seyh Edebali University, 11230 Bilecik, Turkey; 3 Materials Science and Engineering Department, Eskisehir Technical University, 26555 Eskisehir, Turkey; edolekce@eskisehir.edu.tr; 4 Department of Chemistry, Eskisehir Technical University, 26555 Eskisehir, Turkey; merdem@eskisehir.edu.tr; 5 Center for Dental Medicine, Clinic for Masticatory Disorders and Dental Biomaterials, University of Zurich, 8032 Zurich, Switzerland; mutlu.ozcan@zzm.uzh.ch

**Keywords:** dental materials, water sorption, mechanical properties, stearyl methacrylate, prosthodontics

## Abstract

This study evaluated the effect of stearyl methacrylate addition on the physical and mechanical properties of bisphenol A glycidyl methacrylate- and triethylene glycol dimethacrylate-based polymers, which are traditionally used in dental applications. Methacrylate-based monomer compositions are polymerized under the visible blue light spectrum. An analysis of double bond conversion, surface microhardness test, three-point bending test and water sorption and water solubility were tested to determine the physical and mechanical properties of the dental polymers. The results indicated that stearyl methacrylate addition up to 25 wt% reduced the water sorption of the polymers. At amounts of stearyl methacrylate higher than 25 wt%, the solubility of the polymer in water increases due to the monofunctional structure. Mechanical properties are negatively affected by the increasing stearyl methacrylate ratio. Further, the addition of stearyl methacrylate slightly increased thermal stability. As such, the amount of stearyl methacrylate in a polymer composition is critical for the optimization of its mechanical and physical properties. According to the results, the amount of stearyl methacrylate has to be between 12.5–25 wt%.

## 1. Introduction

Tooth hard tissue loss due to dental caries is a common problem in oral health. According to the World Health Organization (WHO), the vast majority of children and all adults worldwide have tooth decay [1]. Teeth play a significant role in human life, both physically and psychologically. Healthy teeth ensure that food is chewed properly, while, on the other hand, good-looking teeth have a positive psychological impact [2]. However, the ongoing increase in life expectancy and changes in eating habits cause dental diseases to increase day by day [3]. Accordingly, the reconstruction of dental hard tissue is becoming more significant for oral rehabilitation. Restorative materials are used to replace the missing hard tissue of the tooth. There is an interest in improving the properties of restorative materials, such as their lifetime, mechanical and aesthetic properties [4]. Composites [5], glass ionomer cements (GICs) [6], resin-modified glass ionomer cements (RMGICs) [7] and polyacid-modified composites (compomers) [8] are dental restorative materials used for different purposes. Compomers contain acidic groups in addition to methacrylate-based monomers. The inorganic component also contains aluminosilicate glasses that can release fluorine. After the restoration process, a limited amount of fluorine is released as a result of the acid–base reaction between the glass component and the polyacid [9]. GICs are an important type of dental restorative material that can continuously release fluorine. However, the colors of GICs are not compatible with the tooth and they wear easily due to their poor mechanical properties. RMGICs have been developed to overcome the poor mechanical and physical properties of GICs. These materials contain monomers in which curing is achieved by photopolymerization, in addition to the curing reaction by acid–base reaction in GICs [10]. The most widely used dental restorative materials are dental composites. Composite materials are preferred for dental restorations instead of amalgams due to their tooth-like appearance and biocompatibility [11]. Dental composites consist of two distinct phases: an organic matrix and an inorganic reinforcement. The organic phase comprises monomer mixtures and the inorganic phase generally comprises ceramic particles. The surface of the ceramic particles is modified with a silane coupling agent to be compatible with the polymer matrix [12]. Additionally, initiation systems are used for the polymerization of matrix monomers [13]. In addition to good mechanical, physical, thermal and tribological properties, dental composites have superior properties, such as their biocompatibility, aesthetic appearance and antibacterial and nontoxic behavior [14].

Various types of dental composites are available commercially. Dental composites can be divided into two groups, as self-curing and light-curing, in terms of curing mechanism and are preferred according to the clinical need [15]. Polymer-based composites curable with light are widely used as a filling for dental restorations. Photopolymerization has become applicable with visible light, which is more environmentally friendly, with the development of UV technology [16]. This development has led to the widespread use of photopolymerization technology in microelectronics, 3D printing, coatings, adhesives and dentistry [17]. In light-cured dental applications, methacrylate-based monomers are generally used as a matrix resin and the resins are reinforced with silane-coupled inorganic materials. Bisphenol A glycidyl methacrylate (Bis-GMA) and triethylene glycol dimethacrylate (TEGDMA) are widely used as resins in commercial dental composites [18]. Each component of the matrix adds a different property to the composite. For example, Bis-GMA provides rigidity and strength, while TEGDMA is used as a diluent [19]. Bis-GMA has high viscosity due to hydroxyl groups and π-π interactions derived from the aromatic ring. Its high viscosity negatively affects the processability of this monomer. For this reason, low-viscosity TEGDMA is added to the composition to reduce viscosity and increase processability [20]. In addition, the high viscosity of Bis-GMA leads to low reaction mobility during photopolymerization [21]. Photopolymerization is the most effective method for the polymerization of methacrylate monomers [22]. Dental composites contain initiator systems for the initiation of photopolymerization. Initiators such as camphorquinone (CQ) are commonly used in methacrylate-based monomers to initiate polymerization with visible light. [23].

Dental composites have many superior advantages, but there are also many challenges to overcome. Dental composite restorations need to be replaced over time for different reasons. These include marginal defects, secondary caries, fractures and discoloration of the restoration material [24]. A number of properties required in dental composites such as low polymerization shrinkage, low water sorption (WS) and water solubility (SL), conversion rate and mechanical strength directly affect the occurrence of these reasons. For example, micro gaps due to improper placement or polymerization shrinkage, or an increase in the surface roughness of the restorative material due to poor mechanical properties, can lead to secondary caries [25]. Marginal defects and fracture are largely related to the mechanical properties of restorative materials. Poor mechanical properties seem to be directly or indirectly related to all of the causes of deterioration of restorative materials. Therefore, the development of aesthetic materials with great mechanical properties has always been the main goal [26]. In addition to providing the necessary properties such as mechanical strength initially, restorative materials must be able to maintain these properties in a moist environment after restoration [27]. WS and SL are among the most important factors that cause the properties of the composite to deteriorate over time. SL is mainly caused by the release of unreacted monomers from composites to the aqueous environment of the mouth. This can lead to increased bacterial growth, allergic reactions and low biocompatibility [28,29]. WS of the composite leads to deterioration of the mechanical properties of composites, and also negatively affects the aesthetic appearance of dental restoratives [30,31,32]. Interactions of polymer chains are broken down due to water uptake [33]. Furthermore, WS makes the polymer chain network more plastic, so wear resistance and chemical stability of dental composite decrease. Polymer matrix–filler interfaces are disrupted via hydrolysis. All these reasons cause deterioration of mechanical properties of dental composites over time [34]. In addition, water molecules of small size can diffuse into nano spaces between chains in polymers [35]. Hydroxy sides of polymer chains react with water, leading to hydrogen bonding. This leads to an increase in water uptake. Because of the hydroxyl groups of Bis-GMA and TEGDMA copolymers, these copolymers behave more hydrophilic, and water uptake of these copolymers is relatively high [36]. 

There are different studies in the literature that reduce the amount of water sorption. In some of the studies, the reduction in water sorption was realized by modification of the inorganic component, while in others the organic component was modified. The use of different reinforcements can reduce the water sorption of the composites [37]. The silanization treatment applied to the reinforcing elements also influences the water sorption behavior. The type and optimum amount of silane compound ensures minimum water sorption [38]. The amount of water sorption in composites also varies depending on the composite production process. Studies have shown that the amount of water sorption increases if the reinforcement process causes voids and decreases if no voids are formed [39,40,41]. It is also seen that the hydrophilicity of the dental composite increases the amount of water sorption of the composite [42,43]. In addition, water sorption and solubility in dental composites are known to be strongly correlated with polymer amount, conversion rate and the amount of unpolymerized monomers [44]. Water sorption is predominantly caused by the matrix component of the composite. Therefore, it is important to carry out water sorption and solubility studies with pure monomers in order to understand their behavior in the composite [18]. In this study, it was aimed to reduce the amount of water sorption by adding a hydrophobic monomer to the polymer composition. Stearyl methacrylate (SMA), which has high hydrophobicity due to its long alkyl chain, was used as a hydrophobic monomer in the study, which has not been tried in dental resins before. The effect of SMA on water sorption, water solubility, flexural strength, flexural modulus and hardness was investigated. Finally, thermogravimetry-differential thermal analysis (TG-DTA) was performed to characterize the thermal behavior of the polymers. SMA also contains double bonds that can react with double bonds of Bis-GMA and TEGDMA [45]. SMA is a mono-functional monomer. Mono-functional monomers lead to the formation of more linear polymers than multifunctional monomers, reducing the amount of crosslinking [46]. Therefore, the addition of a mono-functional monomer causes a decrease in the mechanical properties of the polymer structure obtained. However, secondary interactions arising from the long alkyl chain in the monomers added to the composition are expected to have a positive effect on the mechanical properties. It is thought that the mechanical improvement due to the long alkyl chain of SMA can tolerate the deterioration in mechanical properties caused by mono-functionality.

## 2. Materials and Methods

### 2.1. Materials

The methacrylate monomers used were Bis-GMA (Sigma-Aldrich, St. Louis, MO, USA), TEGDMA (ACROS Organics, Waltham, MA, USA) and SMA (Sigma-Aldrich). All chemicals were used without further purification. CQ (ACROS Organics) 0.5% by weight and Ethyl 4-(dimethyl amino) benzoate (EDMAB) (ACROS Organics) 0.5% by weight were used as the initiator and activator for all compositions.

### 2.2. Preparation of Resins

Four resins were prepared by compositions presented at Table 1. The resins were prepared by the same route. First of all, the Bis-GMA and TEGDMA were stirred mechanically in the determined ratios. Then SMA was added at the weight ratios of 0%, 12.5%, 25% and 37.5% to prepare SMA-0, SMA-12.5, SMA-25 and SMA-37.5, respectively and stirred mechanically. Finally, for the photoactivation CQ and EDMAB were added to the mixture at the weight ratio of 0.5 and stirred mechanically. The whole process was carried out in ambient conditions. The SMA-0 was used as a control specimen. All the specimens were cured by blue light with 400–500 nm wavelengths (Foshan YUYO, LY-B200, 1600 mw/cm^2^, Foshan, Guangdong, China). All of the molds used in sample preparation were made of stainless steel with two open surfaces.

### 2.3. Double Bond Conversion Test

The double bond conversion (DC) values of all the compositions were measured by Fourier transform infrared spectroscopy (FTIR) (Spectrum 100, Perkin Elmer, Waltham, MA, USA) with an attenuated total reflectance (ATR). The FTIR scans were made at 4 cm^−1^ resolution between 650 and 4000 cm^−1^. Firstly, unpolymerized samples were measured for all the compositions. Then, the samples were polymerized for 20 s with a light curing device from both sides of the samples, and FTIR spectrums of the samples were measured immediately after irradiation and one week after irradiation. Samples were kept in the absence of light for one week. Five measurements were performed per sample. DC was determined from the ratio of the aliphatic and aromatic C=C peak intensity at 1636 cm^−1^ and 1608 cm^−1^, respectively. Equation (1) was used for the calculation of DC.

(1) $$DC=(1-\frac{{{\left((C=C\right)}_{Aliphatic}/{\left(C=C\right)}_{Aromatic})}_{After\;Curing}}{{{\left((C=C\right)}_{Aliphatic}/{\left(C=C\right)}_{Aromatic})}_{Before\;Curing}})\times 100,$$

### 2.4. Vickers Microhardness Test

For each composition, four specimens with a diameter of 15 mm and a thickness of 1 mm were prepared. The samples were polymerized with the light curing unit for 20 s from each side of the mold. The microhardness values of each of the specimens were measured after 24 h and 7 days (M1 C010, EMCOTest, Kuchl, Austria). Samples were kept in the absence of light for one week. Five indentations were applied for each specimen and the mean lengths of the indentations were measured. The microhardness was measured with a load of 0.5 kg and 30 s. and obtained with the following Equation (2).

(2) $$HV=\frac{1854.4\times P}{d^{2}},$$

where HV is Vickers hardness (kg/mm^2^), P is the load (grams) and d is the diagonal length of indentations (µm).

### 2.5. Three Point Bending Test

Ten specimens were prepared for all the compositions. A stainless-steel mold with a size of 25 × 2 × 2 mm was filled by mixed pastes. The open faces of the mold were covered with glass microscope slides. The pastes were polymerized with the light curing unit for 20 s from each side of the mold. A 3-point bending test was applied to obtain the flexural strength (FS) and flexural modulus (FM) of the polymerized samples (5581, INSTRON, Norwood, MA, USA). For the 3-point bending test, a 20 mm span and 0.75 mm/min cross-head speed, according to ISO 4049 [47], were used as the test parameters. FS and FM were calculated from the following Equations (3) and (4).

(3) $$FS=\frac{3Fl}{2bd^{2}},$$

(4) $$FM=\frac{Fl^{3}}{4bd^{3}h}$$

where F is the maximum load (N), l is the distances between the supports, b is the width of the specimens, d is the height of the specimens and h is the flexural extension at maximum load.

### 2.6. Water Sorption and Water Solubility Test

The pastes in all compositions were filled into a stainless-steel mold with a diameter of 15 mm and height of 1 mm. The open faces of the mold were covered with glass microscope slides. The pastes were cured for 20 s from each side of the mold. For all compositions, three specimens were prepared. The specimens were dried in a desiccator until a constant mass (m_1_) was obtained at room temperature. Then, the specimens were stored in distilled water for 7 days at 37 °C. The excess water was removed from the surfaces and the masses of specimens were weighed (m_2_). The wet specimens were dried in the desiccator again until a constant mass (m_3_) was obtained at 37 °C. All specimens were weighed at an accuracy of ±0.1 mg. Water sorption (WS) and solubility (SL) were calculated from the following Equations (5) and (6).

(5) $$WS=\frac{m_{2}-m_{3}}{v},$$

(6) $$SL=\frac{m_{1}-m_{3}}{v}$$

where v is the volume in cubic millimeters. The masses are in micrograms. WS and SL are in micrograms per cubic millimeter.

### 2.7. Thermal Analysis Test

Thermal stability and decomposition steps of the samples were analyzed by thermogravimetry-differential thermal analysis (TG-DTA, STA 449F3 Netzsch, Selb, Germany). Thermal analysis was applied with a heating rate of 10 °C/min from 30 to 800 °C temperature range in an air atmosphere with a flow rate of 30 mL/min.

### 2.8. Statistical Analysis

One-way analysis of variance (ANOVA) was used to analyze the test data. Tukey’s post hoc test was applied to determine the differences between the groups. Paired samples *t*-Test was used to determine the differences in microhardness values of the samples after 24 h and 7 days. The statistical significance level was set at α = 0.05 for each analysis.

## 3. Results

### 3.1. Double Bond Conversion

**Figure 1:**
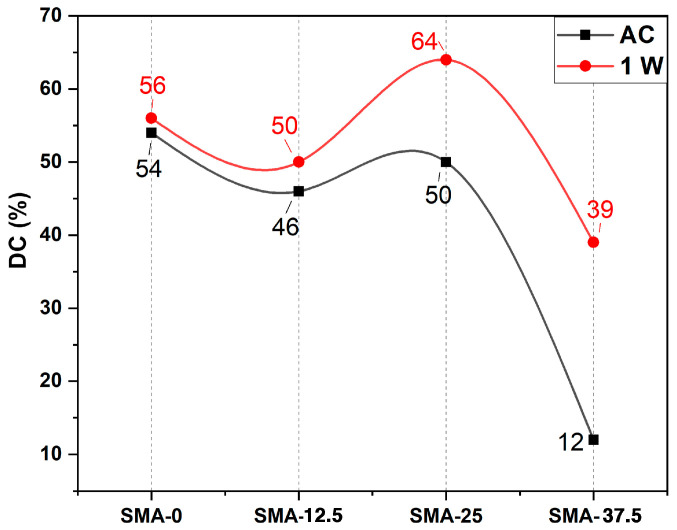
Effect of SMA content on the double bond conversion.

DC values were given in Figure 1. The highest DC value was obtained with SMA-0 after curing. The after-curing DC values of SMA-12.5 and SMA-25 were decreased according to SMA-0, but comparable with SMA-0. The DC value was dramatically decreased at the SMA-37.5 sample. After a week, the DC values of all samples were increased. The highest increase of DC values was obtained in the SMA-37.5. The increases of DC values were 3.7, 8.7, 28 and 225% for SMA-0, SMA-12.5, SMA-25 and SMA-37.5, respectively. After a week, the highest DC value was obtained for SMA-25.

### 3.2. Vickers Microhardness

**Figure 2:**
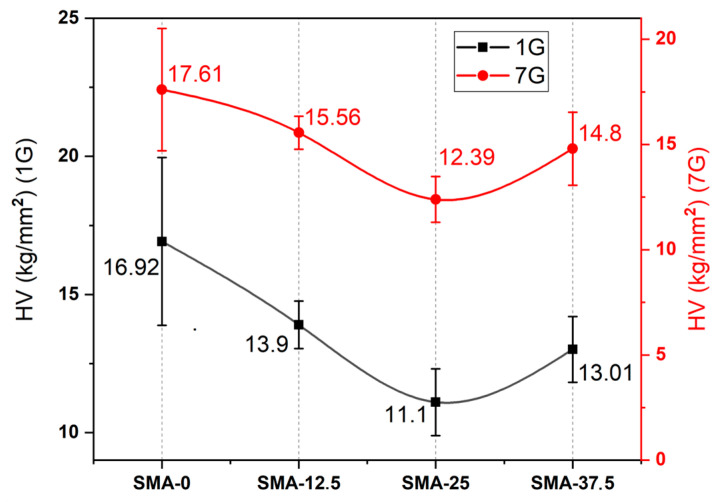
Vickers microhardness (HV) of all the samples.

As shown in Figure 2, when SMA was added to the control sample, the surface microhardness (HV) of the samples decreased (*p* < 0.001). Decreases of HV were around 18–34% and 12–30%, respectively, at 24 h and 7 days, and were not linear. Maximum decreases (34%) of hardness were obtained in the SMA-25. The HV of SMA-0 (*p* > 0.05), SMA-12.5 (*p* < 0.001), SMA-25 (*p* < 0.001) and SMA-37.5 (*p* < 0.05) increased after 7 days compared to 24 h. However, the obtained HV values of SMA-0 after 24 h and 7 days are comparable (*p* > 0.05). The HV of SMA-25 was the lowest (*p* < 0.001) sample among all the samples. SMA-12.5 and SMA-37.5 had comparable HV values (*p* > 0.05).

### 3.3. Three Point Bending

**Figure 3:**
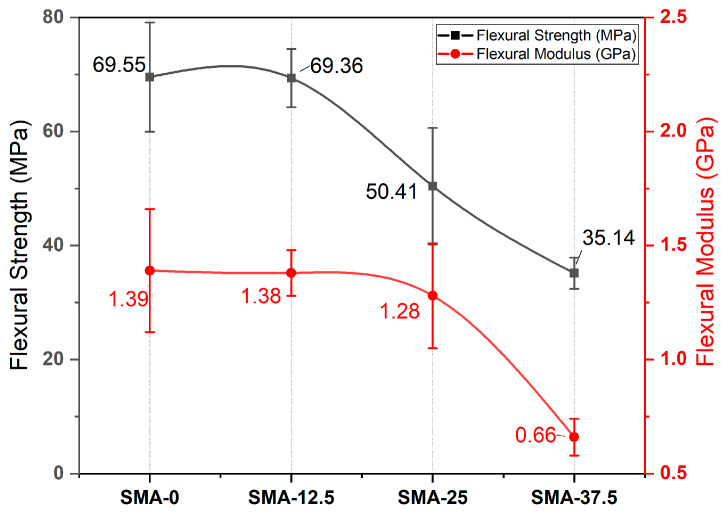
Flexural strength (FS) and flexural modulus (FM) of SMA-0, SMA-12.5, SMA-25 and SMA-37.5.

The results of flexural strength (FS) and flexural modulus (FM) of all samples are given in Figure 3. The FS and FM values of the samples with SMA added were lower than the control sample. The FS and FM of SMA-37.5 with the lowest values (*p* < 0.001) were less than the FS and FM values of the control sample by 49% and 53%, respectively. All of the FS values were statistically different (*p* < 0.001), except for SMA-0 and SMA-12.5 (*p* > 0.05). The FM of SMA-0, SMA-12.5 and SMA-25 were comparable (*p* > 0.05). Only the FM of the SMA-37.5 was statistically different (*p* < 0.001) from the others.

### 3.4. Water Sorption and Water Solubility

**Figure 4:**
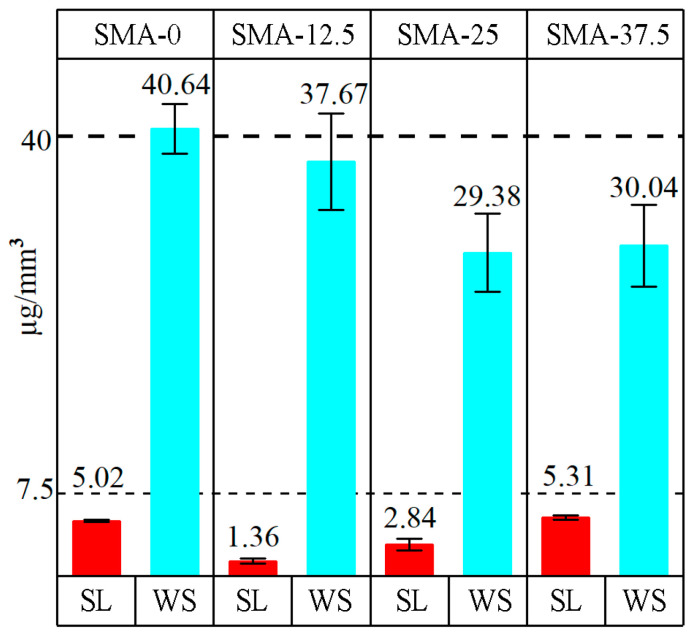
WS and SL of experimental resins: SMA-0, SMA-12.5, SMA-25 and SMA-37.5.

The WS and SL results are summarized in Figure 4. The WS values of samples were decreased with the addition of SMA. When 12.5, 25 and 37.5 wt% SMA was added to the control sample, WS was decreased by 7, 28 and 26%, respectively. The lowest water sorption was obtained at SMA-25. However, the WS of SMA-25 and SMA-37.5 were very similar. SL of SMA-12.5 and SMA-25 decreased 73 and 43%, respectively. The SL of SMA-37.5 increased 6%. The lowest SL was obtained for SMA-12.5, and the SL values of the samples were increased with an increase of SMA quantity.

### 3.5. Thermal Analysis

**Figure 5:**
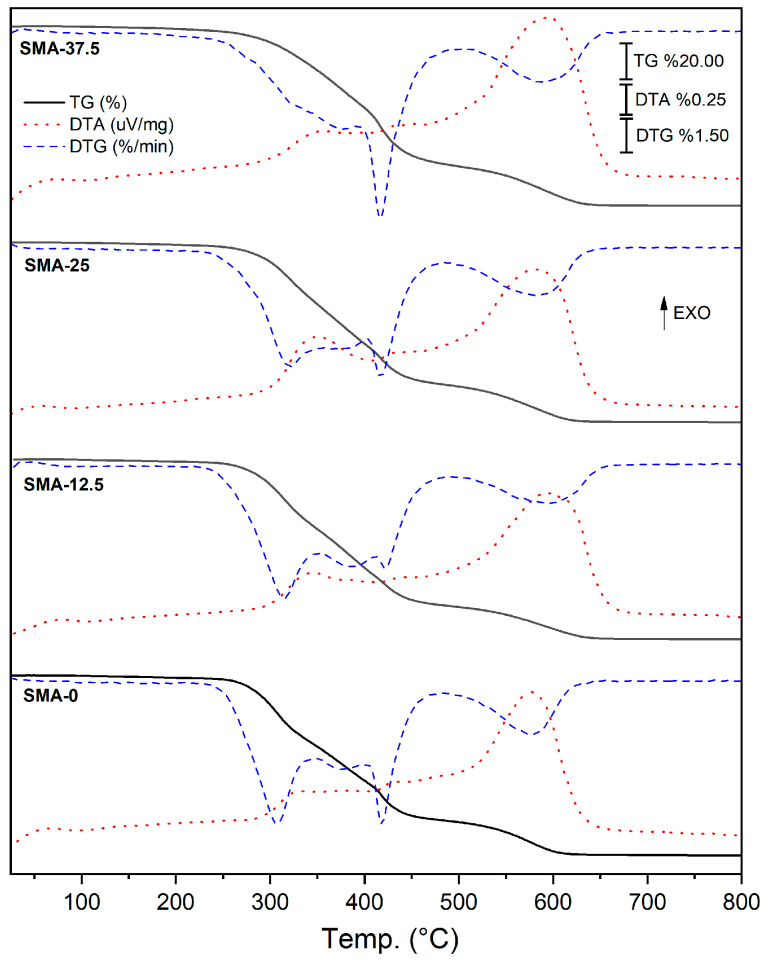
TG/TDA-DTG curves of SMA-0, SMA-12.5, SMA-25 and SMA-37.5.

Figure 5 shows the TG curves of the SMA-0, SMA-12.5, SMA-25 and SMA-37.5 samples. DTG and DTA curves were added to the graphs to better show the mass changes depending on temperature. Details about TG-DTG curves are given in Table 2. The SMA-0, SMA-12.5, SMA-25 and SMA-37.5 samples are stable up to 231, 232, 236 and 242 °C, respectively.

## 4. Discussion

The development of new dental polymers is crucial for maintaining the comfort of life disrupted by dental disease. The longevity of dental restoratives is highly correlated with the stability of the polymers used as matrix elements in dental composites. Additionally, the stability of polymers is closely related to low water sorption. Enhancing hydrophobicity through the addition of a hydrophobic monomer in the polymer formulation is one of the most important ways to decrease water sorption [48]. For this purpose, SMA is added to Bis-GMA–TEGDMA formulations, as shown in Table 1.

SMA is a monofunctional monomer. According to the literature, the presence of monofunctional monomers in bifunctional monomers increases the DC [49,50]. In this study, contrary to the literature, DC values decreased with SMA addition. In our formulation, SMA was added instead of TEGDMA. So, as the amount of SMA increased, the amount of TEGDMA decreased, which resulted in a relatively increased amount of Bis-GMA. This caused a decrease in the DC values. This is because TEGDMA, with low viscosity, enhances the reactivity of monomers due to the increase in mobility of monomers when it is added to Bis-GMA [51,52]. Further, the increase of high molecular weight monomers in polymer compounds negatively affects the DC [53]. It was seen that all the DC values were enhanced after a week. The polymerization reaction occurs in two stages. In the first stage, called auto-acceleration, or the Trommsdorff effect, polymerization is high due to high mobility of monomers. In the second stage, called the glass effect, the polymerization reaction rate decreases due to crosslinking, the reaction being limited by diffusion [46,54,55]. Steric hindrances cause suppression of the first stage as they reduce the polymerization rate [56]. The bulkiness of monomers reduces the polymerization rate at the first stage due to low mobility as well [57]. These reasons lead to the suppression of the first stage during polymerization and the second stage has a greater effect on the DC. The second stage of polymerization can be effective for a longer time, and this is called the post curing mechanism in the literature [58]. In our study, the relatively increased amount of Bis-GMA with a high molecular weight and the bulkiness of SMA monomers resulted in a shortening of the first polymerization stage. Accordingly, the addition of SMA caused a decrease in DC values after curing, especially in the SMA-37.5 sample. Figure 1 shows that as the addition of SMA increases, the amount of increase in DC values after one week also increases. This shows that the post-curing mechanism becomes more effective as the SMA ratio increases. Therefore, the highest DC value increase after one week was obtained in SMA-37.5.

It is clearly seen that the hardness of the samples is decreased with SMA addition. The crosslinking density has a significant effect on the hardness of polymers. It can be said that hardness is a measure of the crosslinking rate [59,60]. Increasing monofunctional monomers in the polymer composition results in a decrease of crosslinking density [61]. Due to the addition of the monofunctional SMA to the polymer composition instead of TEGDMA, the crosslinking density of polymers decreased as expected, and thus the hardness of the polymers decreased. After a week, the hardness of the samples increased. The other effective parameter on hardness is DC [62]. As it can be seen in Figure 1, the DC values of all the samples increased after a week. As such, the crosslinking density and molecular weight of the polymers increased, and therefore the hardness of the samples after a week was higher than the hardness of the samples after curing. According to Figure 2, hardness values tend to decrease. Although it is lower than SMA-0, the hardness of the SMA-37.5 sample has a higher hardness than the SMA-25 sample, unlike its tendency to decrease. At high SMA rates, SMA monomers can polymerize among themselves to form polySMA independently of the Bis-GMA and TEGDMA. Therefore, the polySMA phase can occur in the main polymer matrix, which consists of Bis-GMA, TEGDMA and SMA. This phase can act as reinforcement by restricting the molecular mobility of the matrix polymer. The hardness of SMA-37.5 may have increased as a result of restriction of the polymer chains.

As regards flexural strength and modulus, the values of SMA-0 and SMA-12.5 were statistically similar. The increasing amount of SMA resulted in a decrease in flexural strength and modulus. In this study, the concentrations of Bis-GMA and initiators were kept constant for all samples. The SMA was added instead of TEGDMA. As such, the concentration of TEGDMA decreased with increasing SMA concentration. It is known that the high functionality of monomers causes a high reaction rate and crosslinking density. So, it can be said that monofunctional monomers lead to low flexural strength and modulus [63,64]. The addition of the monofunctional SMA decreased flexural strength and modulus, as expected. The crosslinking density is a critical factor for mechanical properties. At a low crosslinking density, polymers behave more flexibly and show low strength. In our study, the SMA caused low crosslinking density because of its monofunctional structure. Thus, flexural strength and modulus decreased. 

According to Figure 4, the addition of SMA resulted in a decrease in water sorption. Although WS decreased at SMA-12.5 and SMA-25, it increased at SMA-37.5 according to SMA-25, but all values of WS are lower than SMA-0. The SL of the SMA-12.5 decreased with SMA addition. The increase of SMA content resulted in the increase of SL. Blends of Bis-GMA and TEGDMA are accepted as hydrophilic materials with considerably high WS and SL [35]. One of the biggest disadvantages of TEDGMA is its high WS capacity [23]. The amount of WS is related to the chemistry of the polymer composition and the crosslinking density and hydrophilicity of the polymer [34]. WS is low in polymers with high crosslinking density [65]. The addition of monofunctional monomers like SMA to the polymer composition may cause an increase in WS due to decreasing crosslinking density. However, SMA has high hydrophobicity due to the long alkyl chain groups in its structure [45]. Therefore, SMA content at the hydrophilic Bis-GMA TEGDMA polymer increased the hydrophobic behavior of the polymer, although decreasing crosslinking density due to the decreasing TEGDMA amount. When SMA content was increased to 37.5 from 25wt%, WS did not decrease as expected. SMA dissolves in TEGDMA. Increasing SMA with decreasing TEGDMA caused more SMA to be found, unreacted with crosslinking TEGDMA and Bis-GMA monomers. Additionally, the possibility of SMA monomers reacting with each other to form polySMA increases. As can be seen in Figure 4, SL values enhanced with increasing SMA amounts. This may be caused by the release of polySMAs from samples. Increasing SMA simultaneously increased the amount of polySMA, so SL enhanced with increasing SMA amount. Released polySMAs cause micro-voids in the polymer. The lower WS values could not be obtained at the SMA-37.5 sample due to micro voids which occurred related to the release of polySMA.

Figure 5 shows that the addition of SMA slightly increases the thermal stability of the polymers. In all samples, mass loss occurred in three stages. The first stage is the volatilization stage (VS). The mass loss in the VS stage is due to the evaporation of moisture on the sample surface and the combustion of uncured monomers. It is seen that the initial mass loss in the SMA-37.5 sample is higher than the other samples. This is due to the thermal degradation of polySMAs on the surface, which also leads to an increase in the amount of water dissolution. The next stage is the first decomposition (FD) stage. The addition of SMA changes the profile of the FD stage. With the addition of SMA, the mass loss between 300–350 °C in the FD stage decreases, while the mass loss between 400–450 °C increases. In the SMA-37.5 sample, the peak between 300–350 °C almost disappeared. In this sample, the mass loss in the FD stage is mostly above 400 °C. In the SMA-0 sample, it is seen that the mass loss occurs at the beginning of the FD stage. With the increase in the addition of SMA, the effect of SMA, which has a long alkyl chain, on thermal properties increased. It is thought that the addition of SMA monomer to the polymer increases the thermal stability due to the increased physical interactions (Van der Waals bonds) resulting from the long alkyl chain. Therefore, the addition of SMA delays the thermal degradation of the polymer. The last stage is the second decomposition (SD) stage. The mass loss in the SD stage is due to the degradation and oxidation of carbonaceous substances [66].

## 5. Conclusions

The addition of SMA up to 25 wt% to the TEGDMA and Bis-GMA mixture reduced the WS value of the polymers. However, mechanical properties reduced with the increasing SMA ratio due to the monofunctional structure of SMA. According to the results, the amount of SMA is critical for the best composition. For high mechanical properties, the SMA amount has to be lower than 25 wt%, and at the same time, it has to be higher than 12.5 wt% for lower water sorption. It is seen that the addition of SMA slightly increases the thermal stability of the polymer obtained. SMA changes the thermal degradation profile of the polymer.

## Figures and Tables

**Table 1:** Composition of monomers mixtures used in this study.

Abbreviation	Composition of Resins by Weight Percent (%)		
	Bis-GMA	TEGDMA	SMA
SMA-0	50	50	0
SMA-12.5	50	37.5	12.5
SMA-25	50	25	25
SMA-37.5	50	12.5	37.5

**Table 2:** Temperatures and mass loss amounts of thermal stages obtained from TG/DTA-TGA curves.

Samples	Volatilization Step	First Decomposition (FD)	Second Decomposition (SD)	Units
SMA-0	25–231	231–482	482–653	°C
	1.6	78.6	19.8	Δm/%
SMA-12.5	25–232	232–490	490–669	°C
	1.8	79.6	18.6	Δm/%
SMA-25	25–236	236–486	486–676	°C
	1.8	77	21.2	Δm/%
SMA-37.5	25–242	242–508	508–686	°C
	2.7	75.7	21.6	Δm/%

## Data Availability

The data are contained within the article.

## References

[1] Balhaddad A.A., Kansara A.A., Hidan D., Weir M.D., Xu H.H.K., Melo M.A.S. (2019). Toward Dental Caries: Exploring Nanoparticle-Based Platforms and Calcium Phosphate Compounds for Dental Restorative Materials. Bioact. Mater..

[2] Nicholson J., Czarnecka B. (2013). Materials for the Direct Restoration of Teeth.

[3] Bors A., Antoniac I., Cotrut C., Antoniac A., Szekely M. (2016). Surface Analysis of Contemporary Aesthetic Dental Filling Materials after Storage in Erosive Solutions. Mater. Plast..

[4] Esteves R.A., Boaro L.C.C., Gonçalves F., Campos L.M.P., Silva C.M., Rodrigues-Filho L.E. (2018). Chemical and Mechanical Properties of Experimental Dental Composites as a Function of Formulation and Postcuring Thermal Treatment. Biomed. Res. Int..

[5] Bowen R.L. (1956). Use of Epoxy Resins in Restorative Materials. J. Dent. Res..

[6] Wilson A.D., Kent B.E. (1971). The Glass-Ionomer Cement, a New Translucent Dental Filling Material. J. Appl. Chem. Biotechnol..

[7] Wilson A.D., Kent B.E. (1969). Surgical Cement.

[8] Wilson A.D. (1968). Dental Silicate Cements: VII. Alternative Liquid Cement Formers. J. Dent. Res..

[9] McCabe J.F., Walls A. (2008). Applied Dental Materials.

[10] Alsari A., Ghilotti J., Sanz J.L., Llena C., Folguera S., Melo M. (2024). Comparative Evaluation of the Microleakage of Glass Ionomers as Restorative Materials: A Systematic Review of In Vitro Studies. Appl. Sci..

[11] Makvandi P., Jamaledin R., Jabbari M., Nikfarjam N., Borzacchiello A. (2018). Antibacterial Quaternary Ammonium Compounds in Dental Materials: A Systematic Review. Dent. Mater..

[12] Meereis C.T.W., Münchow E.A., de Oliveira da Rosa W.L., da Silva A.F., Piva E. (2018). Polymerization Shrinkage Stress of Resin-Based Dental Materials: A Systematic Review and Meta-Analyses of Composition Strategies. J. Mech. Behav. Biomed. Mater..

[13] Rosa de Lacerda L., Bossardi M., Silveira Mitterhofer W.J., Galbiatti de Carvalho F., Carlo H.L., Piva E., Münchow E.A. (2019). New Generation Bulk-Fill Resin Composites: Effects on Mechanical Strength and Fracture Reliability. J. Mech. Behav. Biomed. Mater..

[14] Yadav R., Kumar M. (2019). Dental Restorative Composite Materials: A Review. J. Oral Biosci..

[15] Guo S., Luo S.H., Li X., Wang Z., Zhang X., Lei X., Yan S.X. (2024). Effect of Silica Nanospheres Silanized by Functional Silanes on the Physicochemical and Mechanical Properties of Bis-GMA/TEGDMA Dental Composite Resin by Photocuring Synthesis. Int. J. Adhes. Adhes..

[16] Sun K., Xiao P., Dumur F., Lalevée J. (2021). Organic Dye-Based Photoinitiating Systems for Visible-Light-Induced Photopolymerization. J. Polym. Sci..

[17] Dumur F. (2022). Recent Advances on Visible Light Pyrrole-Derived Photoinitiators of Polymerization. Eur. Polym. J..

[18] Sideridou I., Tserki V., Papanastasiou G. (2003). Study of Water Sorption, Solubility and Modulus of Elasticity of Light-Cured Dimethacrylate-Based Dental Resins. Biomaterials.

[19] Vouvoudi E.C. (2022). Overviews on the Progress of Flowable Dental Polymeric Composites: Their Composition, Polymerization Process, Flowability and Radiopacity Aspects. Polymers.

[20] Ok I., Aykac A. (2023). Enhancement of the Mechanical and Antibacterial Properties of Bis-GMA/TEGDMA Dental Composite Incorporated with ZnO/CS and Si/PMMA Core–Shell Nanostructures. Chem. Pap..

[21] Ma Z., Chen Y., Wang R., Zhu M. (2024). Synthesis of Polymerizable Betulin Maleic Diester Derivative for Dental Restorative Resins with Antibacterial Activity. Dent. Mater..

[22] Bouzidi A., Bayou S., Khier N., Dehamchia M. (2024). Photoinitiated Polymerization of a Dental Formulation, Part 2: Kinetic Studies. Polym. Bull..

[23] Pratap B., Gupta R.K., Bhardwaj B., Nag M. (2019). Resin Based Restorative Dental Materials: Characteristics and Future Perspectives. Jpn. Dent. Sci. Rev..

[24] Eltahlah D., Lynch C.D., Chadwick B.L., Blum I.R., Wilson N.H.F. (2018). An Update on the Reasons for Placement and Replacement of Direct Restorations. J. Dent..

[25] Bhadila G.Y., Baras B.H., Balhaddad A.A., Williams M.A., Oates T.W., Weir M.D., Xu H.H.K. (2023). Recurrent Caries Models to Assess Dental Restorations: A Scoping Review. J. Dent..

[26] Albergaria L.S., Scotti C.K., Mondelli R.F.L., Vega H.A., Faggion C.M., Bombonatti J.F.S., Velo M.M. (2023). de A.C. Effect of Nanofibers as Reinforcement on Resin-Based Dental Materials: A Systematic Review of in Vitro Studies. Jpn. Dent. Sci. Rev..

[27] Podgórski M., Becka E., Claudino M., Flores A., Shah P.K., Stansbury J.W., Bowman C.N. (2015). Ester-Free Thiol-Ene Dental Restoratives—Part B: Composite Development. Dent. Mater..

[28] Sideridou I., Achilias D.S., Spyroudi C., Karabela M. (2004). Water Sorption Characteristics of Light-Cured Dental Resins and Composites Based on Bis-EMA/PCDMA. Biomaterials.

[29] Lai J.Y., Wang T.P., Li Y.T., Tu I.H. (2012). Synthesis, Characterization and Ocular Biocompatibility of Potential Keratoprosthetic Hydrogels Based on Photopolymerized Poly(2-Hydroxyethyl Methacrylate)-Co-Poly(Acrylic Acid). J. Mater. Chem..

[30] Kim J.G., Chung C.M. (2003). Trifunctional Methacrylate Monomers and Their Photocured Composites with Reduced Curing Shrinkage, Water Sorption, and Water Solubility. Biomaterials.

[31] van Noort R. (2013). Introduction to Dental Materials.

[32] Benkeser S.M., Karlin S., Rohr N. (2024). Effect of Curing Mode of Resin Composite Cements on Water Sorption, Color Stability, and Biaxial Flexural Strength. Dent. Mater..

[33] Fugolin A.P., Dobson A., Mbiya W., Navarro O., Ferracane J.L., Pfeifer C.S. (2019). Use of (Meth)Acrylamides as Alternative Monomers in Dental Adhesive Systems. Dent. Mater..

[34] Aminoroaya A., Neisiany R.E., Khorasani S.N., Panahi P., Das O., Madry H., Cucchiarini M., Ramakrishna S. (2021). A Review of Dental Composites: Challenges, Chemistry Aspects, Filler Influences, and Future Insights. Compos. B Eng..

[35] Ito S., Hashimoto M., Wadgaonkar B., Svizero N., Carvalho R.M., Yiu C., Rueggeberg F.A., Foulger S., Saito T., Nishitani Y. (2005). Effects of Resin Hydrophilicity on Water Sorption and Changes in Modulus of Elasticity. Biomaterials.

[36] Sankarapandian M., Shobha H., Kalachandra S., Mcgrath J. (1997). Characterization of Some Aromatic Dimethacrylates for Dental Composite Applications. J. Mater. Sci. Mater. Med..

[37] Shekofteh K., Kashi T.J., Behroozibakhsh M., Sadr A., Najafi F., Bagheri H. (2023). Evaluation of Physical/Mechanical Properties of an Experimental Dental Composite Modified with a Zirconium-Based Metal-Organic Framework (MOF) as an Innovative Dental Filler. Dent. Mater..

[38] Varghese J.T., Cho K., Raju, Farrar P., Prentice L., Prusty B.G. (2023). Effect of Silane Coupling Agent and Concentration on Fracture Toughness and Water Sorption Behaviour of Fibre-Reinforced Dental Composites. Dent. Mater..

[39] Saini S., Meena A., Yadav R., Patnaik A. (2023). Investigation of Physical, Mechanical, Thermal, and Tribological Characterization of Tricalcium Phosphate and Zirconia Particulate Reinforced Dental Resin Composite Materials. Tribol. Int..

[40] Li H., Huang J., Zhang H., Hang R., Wang Y. (2024). Preparation of Al-Doped Mesoporous Silica Spheres (Al-MSSs) for the Improvement of Mechanical Properties and Aging Resistance of Dental Resin Composites. J. Mech. Behav. Biomed. Mater..

[41] Althahban S., Alomari A.S., Sallam H.E.-D.M., Jazaa Y. (2023). An Investigation of Wear, Mechanical, and Water Sorption/Solubility Behaviors of a Commercial Restorative Composite Containing Nano-Additives. J. Mater. Res. Technol..

[42] Tihan T.G., Ionita M.D., Popescu R.G., Iordachescu D. (2009). Effect of Hydrophilic-Hydrophobic Balance on Biocompatibility of Poly(Methyl Methacrylate) (PMMA)-Hydroxyapatite (HA) Composites. Mater. Chem. Phys..

[43] Örtengren U., Wellendorf H., Karlsson S., Ruyter I.E. (2001). Water Sorption and Solubility of Dental Composites and Identification of Monomers Released in an Aqueous Environment. J. Oral Rehabil..

[44] Thanoon H., Silikas N., Watts D.C. (2024). Effect of Polymerisation Protocols on Water Sorption, Solubility and Hygroscopic Expansion of Fast-Cure Bulk-Fill Composite. Dent. Mater..

[45] Rattanawongwiboon T., Haema K., Pasanphan W. (2014). Stearyl Methacrylate-Grafted-Chitosan Nanoparticle as a Nanofiller for PLA: Radiation-Induced Grafting and Characterization. Radiat. Phys. Chem..

[46] Atai M., Watts D.C., Atai Z. (2005). Shrinkage Strain-Rates of Dental Resin-Monomer and Composite Systems. Biomaterials.

[47] (2019). Dentistry—Polymer-Based Restorative Materials.

[48] Yiu C.K.Y., King N.M., Carrilho M.R.O., Sauro S., Rueggeberg F.A., Prati C., Carvalho R.M., Pashley D.H., Tay F.R. (2006). Effect of Resin Hydrophilicity and Temperature on Water Sorption of Dental Adhesive Resins. Biomaterials.

[49] Rueggeberg F., Tamareselvy K. (1995). Resin Cure Determination by Polymerization Shrinkage. Dent. Mater..

[50] Asmussen E., Peutzfeldt A. (2001). Influence of Selected Components on Crosslink Density in Polymer Structures. Eur. J. Oral Sci..

[51] Asmussen E. (1982). Factors Affecting the Quantity of Remaining Double Bonds in Restorative Resin Polymers. Eur. J. Oral Sci..

[52] Ferracane J.L., Greener E.H. (1986). The Effect of Resin Formulation on the Degree of Conversion and Mechanical Properties of Dental Restorative Resins. J. Biomed. Mater. Res..

[53] Alabdali Z.N., Reiter M.P., Lynch-Branzoi J.K., Mann A.B. (2021). Compositional Effects on Mechanical Properties and Viscosity in UDMA-MMA Blends. J. Adhes. Sci. Technol..

[54] Fugolin A.P., de Paula A.B., Dobson A., Huynh V., Consani R., Ferracane J.L., Pfeifer C.S. (2020). Alternative Monomer for BisGMA-Free Resin Composites Formulations. Dent. Mater..

[55] Suzuki Y., Mishima R., Kato E., Matsumoto A. (2023). Analysis of the Glass Effect and Trommsdorff Effect during Bulk Polymerization of Methyl Methacrylate, Ethyl Methacrylate, and Butyl Methacrylate. Polym. J..

[56] González-López J.A., Pérez-Mondragón A.A., Cuevas-Suárez C.E., Trejo-Carbajal N., Herrera-González A.M. (2020). Evaluation of Dental Composites Resins Formulated with Non-Toxic Monomers Derived from Catechol. J. Mech. Behav. Biomed. Mater..

[57] He J., Liu F., Luo Y., Jia D. (2012). Synthesis and Characterization of a Dimethacrylates Monomer with Low Shrinkage and Water Sorption for Dental Application. J. Appl. Polym. Sci..

[58] Aromaa M.K., Vallittu P.K. (2018). Delayed Post-Curing Stage and Oxygen Inhibition of Free-Radical Polymerization of Dimethacrylate Resin. Dent. Mater..

[59] Pérez-Mondragón A.A., Cuevas-Suárez C.E., González-López J.A., Trejo-Carbajal N., Meléndez-Rodríguez M., Herrera-González A.M. (2020). Preparation and Evaluation of a BisGMA-Free Dental Composite Resin Based on a Novel Trimethacrylate Monomer. Dent. Mater..

[60] Hwang H.D., Park C.H., Moon J.I., Kim H.J., Masubuchi T. (2011). UV-Curing Behavior and Physical Properties of Waterborne UV-Curable Polycarbonate-Based Polyurethane Dispersion. Prog. Org. Coat..

[61] Liu H., Lin F., Chen M., Xu K. (2010). Preparation and Properties of New UV-Curable Naphtyl Epoxy Acrylates. Iran. Polym. J..

[62] Szczesio-Wlodarczyk A., Domarecka M., Kopacz K., Sokolowski J., Bociong K. (2021). An Evaluation of the Properties of Urethane Dimethacrylate-Based Dental Resins. Materials.

[63] He J., Garoushi S., Säilynoja E., Vallittu P.K., Lassila L. (2019). The Effect of Adding a New Monomer “Phene” on the Polymerization Shrinkage Reduction of a Dental Resin Composite. Dent. Mater..

[64] Wang X., Soucek M.D. (2013). Investigation of Non-Isocyanate Urethane Dimethacrylate Reactive Diluents for UV-Curable Polyurethane Coatings. Prog. Org. Coat..

[65] Kerby R.E., Knobloch L.A., Schricker S., Gregg B. (2009). Synthesis and Evaluation of Modified Urethane Dimethacrylate Resins with Reduced Water Sorption and Solubility. Dent. Mater..

[66] Da Silva Tanaka A.C., Dos Santos G.C., Alarcon R.T., Da Silva Filho L.C., Rezende M.C.R.A. (2021). Effect of the Incorporation of EDB Co-Initiator in the Resin in Halogen and LED Light. Mater. Res..

